# X-ray Findings in a Case of Acute Osteomyelitis Following Puncture Injury: A Case Report

**DOI:** 10.7759/cureus.104291

**Published:** 2026-02-26

**Authors:** Cameron Juybari, Darby Beeson, Emmelyn Samones, Robert M Allison

**Affiliations:** 1 Emergency Medicine, Loma Linda University Medical Center, Loma Linda, USA; 2 Emergency Medicine, Loma Linda University School of Medicine, Loma Linda, USA

**Keywords:** case report, diabetic infection, methicillin-resistant staphylococcus aureus, osteomyelitis, puncture wound

## Abstract

Puncture wounds are common injuries that may lead to infection. Diabetic patients are at higher risk for serious infections, including osteomyelitis. Osteomyelitis is an infection of the bone that requires a combination of surgical and antibiotic therapy. X-ray findings of osteomyelitis include demineralization, periosteal reaction, and bony destruction. This case follows a 62-year-old woman with a history of uncontrolled type 2 diabetes mellitus who presented to the emergency department (ED) for left foot pain after she stepped on a nail that punctured her foot through her shoe. Four days before presentation, she was evaluated at an outside ED and prescribed cephalexin for foot cellulitis. She returned to the ED with worsening foot pain and swelling despite compliance with cephalexin. A foot X-ray was obtained and demonstrated evidence of acute osteomyelitis. The diagnosis was confirmed with magnetic resonance imaging and, ultimately, a bone biopsy. The patient received intravenous antibiotics and surgical ray amputation. At follow-up, she showed clinical improvement, with notable wound healing and reduced symptoms. The primary takeaways from this case are the importance of considering expanding workups for patients who return to the ED and that X-rays can provide important diagnostic information for acute osteomyelitis, despite not having a high sensitivity.

## Introduction

Puncture wounds are a common type of injury that can become complicated by infection [1,2]. The injury occurs when a sharp object, such as a nail, penetrates the skin barrier and enters the underlying fat and muscle. Due to the seemingly trivial nature of the injury, many do not seek medical care; thus, the number of infections secondary to puncture injuries is likely underreported [3]. A survey of emergency room patients regarding plantar puncture wounds revealed that 44% of respondents had at least one plantar puncture wound, and 50% of these wounds were seen by a physician [3]. The infection rate for plantar puncture wounds is estimated at 6%-11% [2]. These soft-tissue infections are usually caused by gram-positive organisms, including Staphylococcal species (most commonly *Staphylococcus* *aureus*) and Streptococcal species. *Pseudomonas aeruginosa* infection typically occurs from a puncture injury through the sole of a shoe [2]. Cellulitis can occur when a penetrating object violates the dermis and subcutaneous tissue [1,2]. Furthermore, penetration into the bone or periosteum may lead to osteomyelitis [1,2].

Twenty percent of all diabetic-related admissions are due to foot complications [4]. Diabetic foot complications are the most common reason for nontraumatic amputations [4]. Osteomyelitis is an infection of the bone. Osteomyelitis arises from either hematogenous seeding, extension from nearby infection, or direct inoculation [5]. In young adults, the most common cause is trauma and open fractures, whereas in pediatrics and geriatrics, the most common cause is bacteremia [6]. Osteomyelitis typically arises from chronic ulcers in diabetic patients [5]. Sixty percent of diabetic foot ulcers are associated with underlying infections [7]. Inadequate treatment of osteomyelitis increases the risk for amputation [4]. Diagnosis requires two of the four following criteria: 1) purulent drainage from the wound site, 2) positive bone biopsy or blood culture, 3) localized physical exam findings of bone tenderness and edema, and 4) radiographical evidence [6]. Radiographical evidence can be found on X-ray, computed tomography (CT), and/or magnetic resonance imaging (MRI) [6-8]. Osteomyelitis is managed with intravenous (IV) antibiotics and referral to surgery for potential amputation and referral to infectious disease [6].

## Case presentation

A 62-year-old woman with a history of type 2 diabetes mellitus (last measured hemoglobin A1c was 11.1% one month prior to presentation), a left diabetic foot wound requiring second digit amputation five years ago, non-ST-elevation myocardial infarction, hyperlipidemia, and hypertension presented for left foot pain. She reported a penetrating injury to her foot five days prior to emergency department (ED) presentation. The patient stated that she stepped on a nail while wearing shoes and endorsed foot pain and swelling that started three days later. She denied fever. She initially presented to an outside hospital, where she was diagnosed with cellulitis, prescribed cephalexin, and administered a tetanus booster. Vital signs on presentation to our ED include blood pressure 147/52 mmHg, heart rate 78 bpm, temperature 97.4°F, respirations 16 breaths/minute, and oxygen saturation 100% on room air.

**Figure 1:**
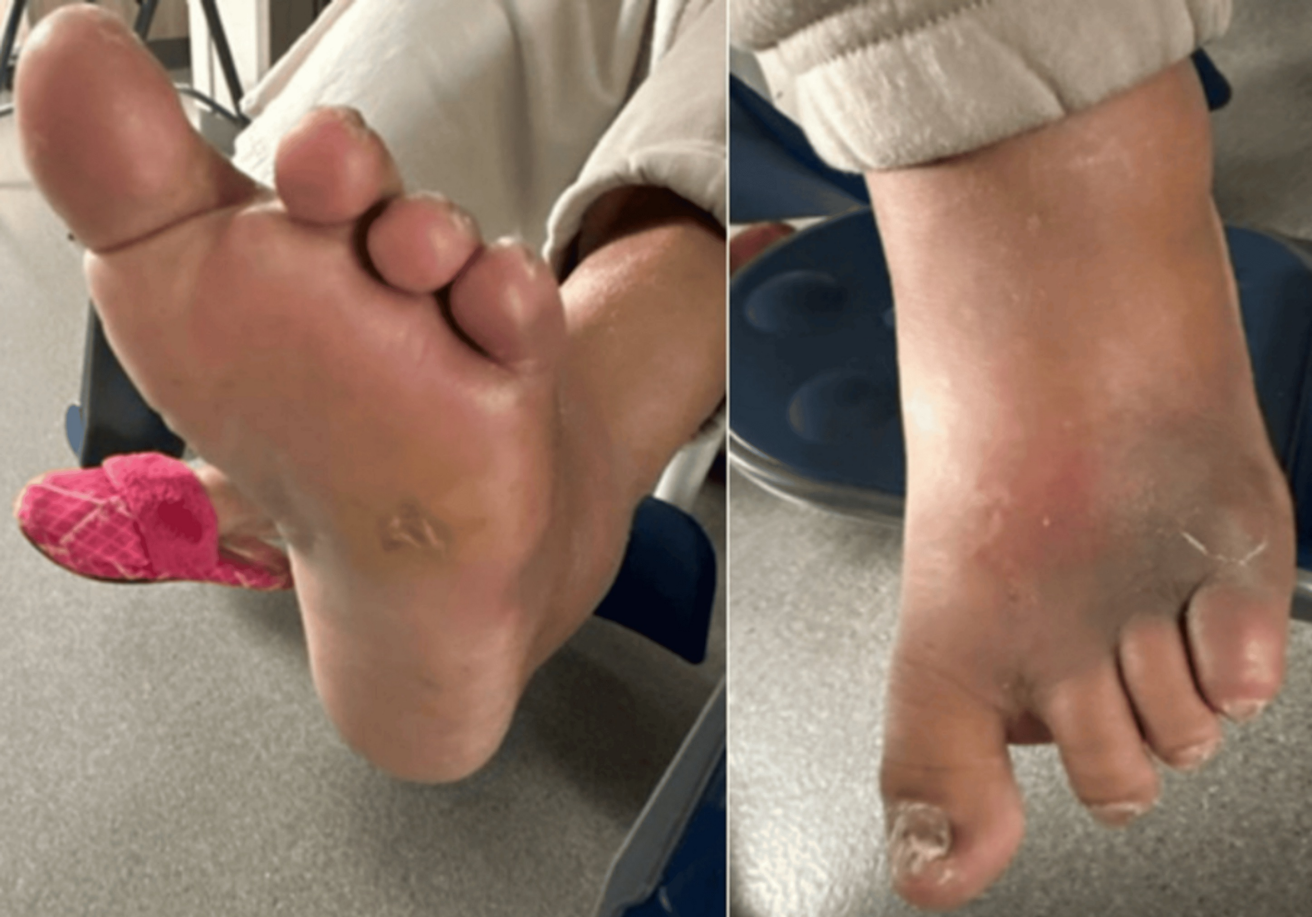
Diabetic left foot wound five days after penetrating injury

**Figure 2:**
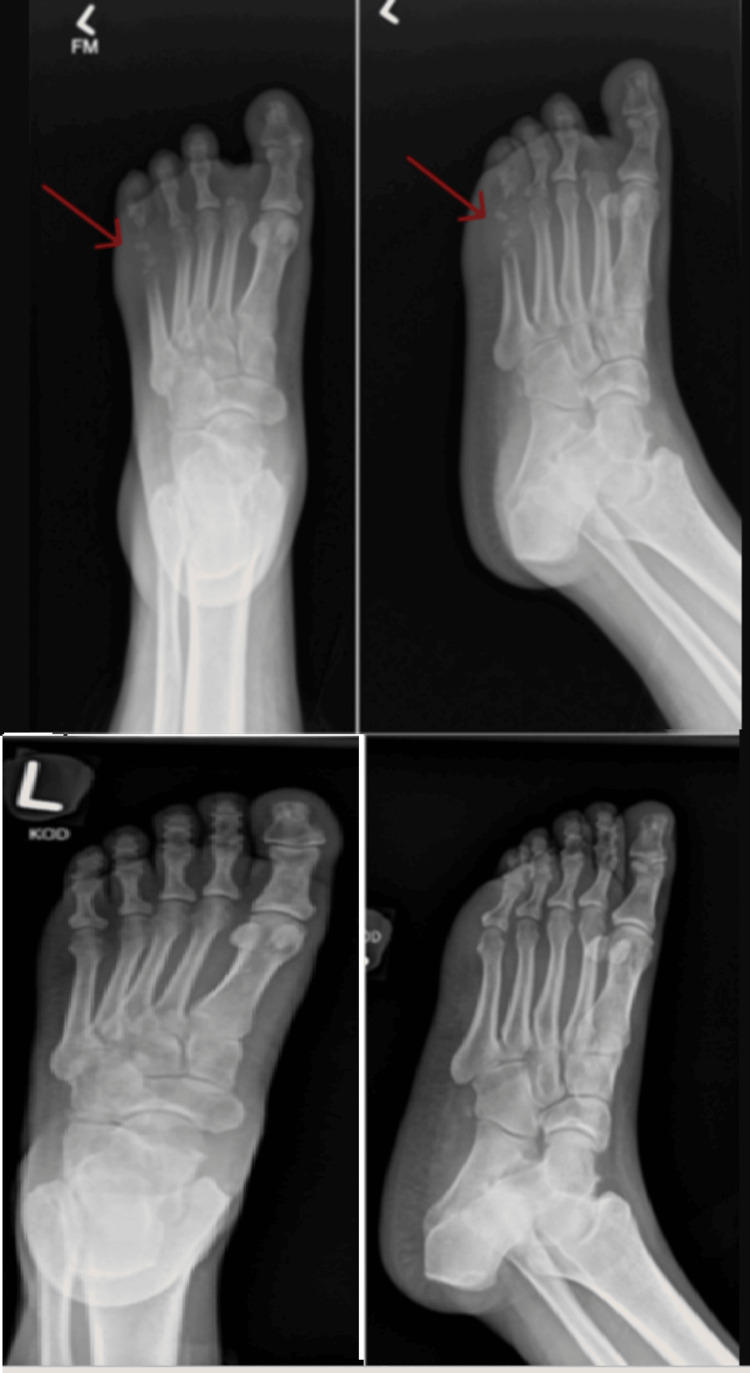
Radiographs of the left foot, demonstrating osteolysis of the fifth distal metatarsal with probable extension to the distal phalanx concerning for acute osteomyelitis (top row, arrow). Radiographs of the left foot for comparison from nine years before (bottom row)

Physical examination was significant for a swollen, warm, purple left foot without crepitus (Figure 1). Labs were significant for elevated glucose of 205 mg/dL (reference range: 70-140 mg/dL) and C-reactive protein (CRP) of 10.9 mg/L (reference range: <1.0 mg/L). Blood cultures were drawn and eventually resulted in no growth. Radiograph of the left foot demonstrated osteolysis of the fifth distal metatarsal with probable extension to the distal phalanx concerning for acute osteomyelitis (Figure 2). A CT of the foot demonstrated evidence of osteomyelitis as well as evidence of septic arthritis.

The case was discussed with an orthopedic surgeon, who recommended admission to internal medicine for IV antibiotics without acute surgical intervention. The patient was started on IV vancomycin and piperacillin-tazobactam. The admitting team consulted general surgery, and they recommended internal medicine admission, an MRI of the foot, and IV antibiotics. Results of an MRI of the left foot obtained on day 1 of hospitalization included the following. There is osseous destruction and cortical discontinuity centered in the fifth metatarsophalangeal (MTP) joint. There is also osseous edema and loss of fat marrow signal involving the fifth toe mid to distal phalanges, and there is a rim-enhancing fluid collection centered in the fifth MTP joint measuring 1.5 cm transverse by 2.3 cm anteroposterior. The impression is that osteomyelitis is extending from the fifth metatarsal to the distal phalanges, with worse involvement at the level of the fifth MTP joint, and possible superimposed fracture. The associated abscess measures up to 2.3 cm. The MRI findings of osseous edema, loss of fat marrow signal, and abscess are suggestive of acute osteomyelitis rather than a more chronic process.

**Figure 3:**
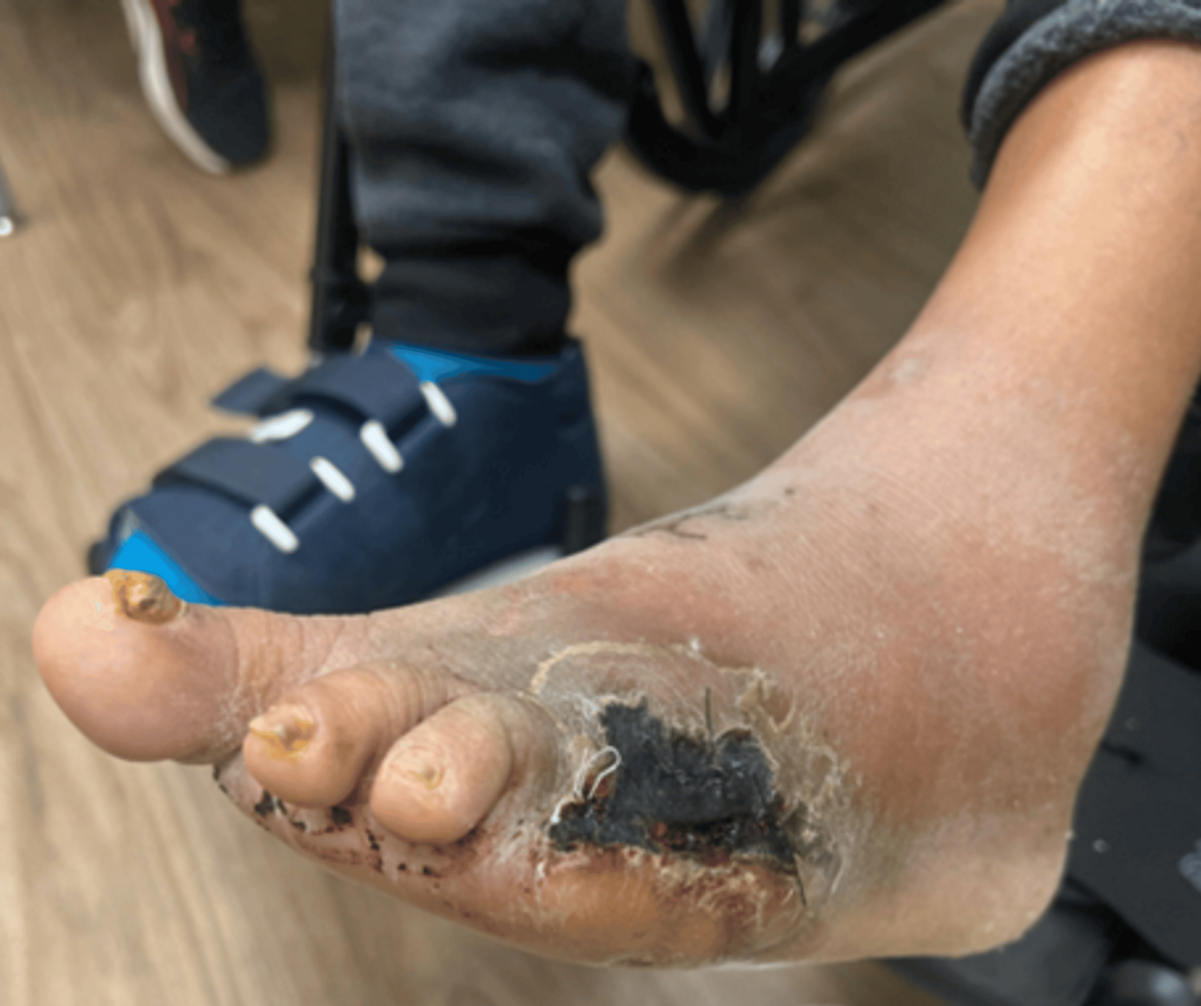
Diabetic left foot wound complicated by osteomyelitis and abscess, status post-incision and drainage with IV antibiotic therapy, and status post-ray amputation of the fifth digit IV: intravenous

On day 2 of admission, orthopedic surgery performed a bone biopsy, which was speciated on day 4 of admission and grew methicillin-resistant *S. aureus* (MRSA). On day 3 of admission, the general surgery team performed an incision and drainage of the abscess and ray amputation of the fifth digit. Wound cultures from the operation speciated on day 5 of admission and grew MRSA, *Staphylococcus* ​​​​​​*simulans, *and *Paenibacillus timonensis*. Infectious disease recommended six weeks of IV daptomycin and doxycycline via peripherally inserted central catheter after source control was achieved. The patient was ultimately discharged on postoperative day 5. On day 8 after discharge, she was seen by her primary care provider, who noted no erythema of the foot and only scant blood from her wound (Figure 3).

## Discussion

Our patient had laboratory and radiographic testing that yielded the diagnosis of osteomyelitis. The appropriate specialists, including infectious disease, orthopedics, and general surgery, were consulted. The patient underwent the recommended treatment with antibiotics and surgical intervention, and improvement was noted in her hospital follow-up appointment.

Interestingly, historical components relating to the wound are poorly studied in diabetic foot wounds in their association with osteomyelitis [4]. The physical exam findings that increase the likelihood are a diabetic foot ulcer area greater than 2 cm^2^ and positive probe to bone testing [4]. Our patient did not have an ulcer. Diabetic patients have a higher likelihood of having osteomyelitis develop from puncture wounds [9]. Additionally, one study found that 35% of diabetic patients admitted for diabetic foot infection had osteomyelitis, and 81.5% of these patients had polymicrobial osteomyelitis [9]. Based on this study, our patient is already at a higher risk for osteomyelitis from having a seemingly infected puncture wound.

In this case report, an X-ray was the first radiographic test performed, and it showed evidence of bone destruction of the fifth digit. A negative radiograph does not provide enough diagnostic certainty to rule out osteomyelitis, as 95% of films are within normal limits at presentation [6]. After 28 days of infection, 90% of films show some evidence of changes [6]. This case is unique because classical radiograph findings were already present by day 5 of symptom onset. Classical X-ray findings include demineralization, periosteal reaction, and bony destruction [7]. Although X-rays can provide rapid diagnostic evidence, their utility is limited in the diagnosis of osteomyelitis [7,10]. The sensitivity of X-ray to diagnose osteomyelitis in diabetic foot wounds is 54%, while the specificity is 68% [10]. Despite the low diagnostic accuracy of X-ray for osteomyelitis, X-ray remains a crucial component as a screening tool, as it is rapid and inexpensive [7]. Furthermore, it can identify free air, suggesting a necrotizing soft tissue infection, which changes the immediate management [7].

The patient had a normal white blood cell count. Fifty-six percent of patients with diabetic foot wounds have normal leukocyte counts [7]. The current literature regarding leukocytosis in diabetic osteomyelitis is mixed, and the consensus is that it is not a reliable diagnostic marker [7]. CRP, a marker of inflammation, tends to be elevated in infected diabetic foot wounds [7]. A CRP level greater than 3.2 mg/dL was demonstrated to be associated with a sensitivity of 85% and specificity of 65% in the diagnosis of osteomyelitis in the presence of a diabetic foot ulcer [7,11]. The CRP in this case report is also similarly elevated.

MRI sensitivity has been reported to range from 77% to 100%, and specificity from 40% to 100%. The authors of this meta-analysis conclude that it can be used to rule in or out the diagnosis of osteomyelitis [7,12]. Bone biopsy is often required to identify etiological pathogens to guide clinical treatment [5]. It is the gold standard in diagnosis [4]. CT scan lacks sensitivity for acute osteomyelitis but provides diagnostic information on other components of soft-tissue infection, such as abscesses and necrotizing infections [6,8]. Our patient had positive radiographic evidence on all three imaging modalities, a positive bone biopsy, and surgical cultures.

## Conclusions

This case underscores the critical importance of broadening the differential diagnosis and work-up in patients with soft-tissue infections who fail to improve with outpatient antibiotic therapy. Furthermore, due to the high prevalence of *P.* *aeruginosa *infection from puncture wounds through shoes, the patient may have benefited from broader coverage initially, as cephalexin does not provide excellent coverage for *P. aeruginosa*. Ciprofloxacin is classically recommended for these types of wounds; however, in our case, as the bone biopsy demonstrated MRSA, ciprofloxacin may not have ultimately provided adequate coverage.

## References

[1] Laughlin RT, Reeve F, Wright DG, Mader JT, Calhoun JH (1997). Calcaneal osteomyelitis caused by nail puncture wounds. Foot Ankle Int.

[2] Quinn J (2020). Puncture wounds and bites. Tintinalli's Emergency Medicine: A Comprehensive Study Guide.

[3] Weber EJ (1996). Plantar puncture wounds: a survey to determine the incidence of infection. J Accid Emerg Med.

[4] Butalia S, Palda VA, Sargeant RJ, Detsky AS, Mourad O (2008). Does this patient with diabetes have osteomyelitis of the lower extremity?. JAMA.

[5] Miller JM, Binnicker MJ, Campbell S (2018). A guide to utilization of the microbiology laboratory for diagnosis of infectious diseases: 2018 update by the Infectious Diseases Society of America and the American Society for Microbiology. Clin Infect Dis.

[6] Fayad LM, Carrino JA, Fishman EK (2007). Musculoskeletal infection: role of CT in the emergency department. Radiographics.

[7] Dinh T, Snyder G, Veves A (2010). Current techniques to detect foot infection in the diabetic patient. Int J Low Extrem Wounds.

[8] Pierce JL, Perry MT, Wessell DE (2022). ACR Appropriateness Criteria® suspected osteomyelitis, septic arthritis, or soft tissue infection (excluding spine and diabetic foot): 2022 update. J Am Coll Radiol.

[9] Lavery LA, Walker SC, Harkless LB, Felder-Johnson K (1995). Infected puncture wounds in diabetic and nondiabetic adults. Diabetes Care.

[10] Hingorani A, LaMuraglia GM, Henke P (2016). The management of diabetic foot: a clinical practice guideline by the Society for Vascular Surgery in collaboration with the American Podiatric Medical Association and the Society for Vascular Medicine. J Vasc Surg.

[11] Fleischer AE, Didyk AA, Woods JB, Burns SE, Wrobel JS, Armstrong DG (2009). Combined clinical and laboratory testing improves diagnostic accuracy for osteomyelitis in the diabetic foot. J Foot Ankle Surg.

[12] Kapoor A, Page S, Lavalley M, Gale DR, Felson DT (2007). Magnetic resonance imaging for diagnosing foot osteomyelitis: a meta-analysis. Arch Intern Med.

